# Evaluation of surgical antimicrobial prophylaxis practices in a tertiary care center in Oman, 2015

**DOI:** 10.1186/2047-2994-4-S1-P177

**Published:** 2015-06-16

**Authors:** WI El-Shoubary, M Elsheikh, A Al Maani

**Affiliations:** 1 Directorate General for Disease Surveillance and Control, Department of Infection Prevention and Control, Ministry of Health, Oman, Muscat, Oman

## Introduction

Surgical Antimicrobial Prophylaxis (SAP) is currently considered an essential component of the standard of care in virtually all surgical procedures. It has resulted in reduced post-operative morbidity and mortality. National guidelines for SAP is lacking in Oman.

## Objectives

This study evaluates the practices of surgeons regarding SAP in a tertiary care center in Oman, to identify gaps, and to set recommendations.

## Methods

Prospective, observational study. All patients from all surgical departments, who had undergone elective, clean or clean contaminated surgery over a 2-month period in 2015, were followed up till discharge from the center. The number & types of antimicrobials used along with duration were noted, described, and compared.

## Results

From a total of 478 patients enrolled in the study, 465 (97%) patients received antimicrobials for therapeutic, prophylactic, or both reasons (5%, 71%, and 21% respectively). The antimicrobials used for SAP varied among different departments but, generally, cefuroxime was the most commonly used antimicrobial (58%), followed by amoxicillin/clavulanic acid (21%), then mitronidazole (15%). Most of SAP (74%) were given within one hour of incision in agreement with the international guidelines. However, inappropriate prolonged duration, of SAP administration, for more than 24 hours were noted in 69% of patients. There was a significant difference among different surgical departments regarding timing and duration of SAP (p<0.01), while there was no significant difference between the clean and clean contaminated groups.

## Conclusion

There is a great variability in SAP practices among different surgical departments inside the same center. A national guideline for SAP is urgently needed to help standardizing practices. Post implementation auditing of SAP guidelines will help evaluating the compliance of surgical team and its impact on the patients’ morbidity and mortality as well as on the overall use of antimicrobials.

## Disclosure of interest

None declared.

